# Induction of Pyroptosis in Renal Tubular Epithelial Cells Using High Glucose

**DOI:** 10.3389/fmed.2022.874916

**Published:** 2022-05-27

**Authors:** Yinghong Liu, Mingyue He, Hao Xiong, Fang Yuan

**Affiliations:** Hunan Key Laboratory of Kidney Disease and Blood Purification, Department of Nephrology, The Second Xiangya Hospital of Central South University, Changsha, China

**Keywords:** diabetic kidney disease, GSDMD, NLRP3 inflammasome, pyroptosis, renal tubular epithelial cells

## Abstract

**Background:**

The micro-inflammatory state is important for the occurrence of diabetic kidney disease (DKD). Here, we aimed to explore the expression of pyroptosis related indicators and ultrastructural characteristics in DKD, and investigate pyroptosis in renal tubular epithelial cells induced by high glucose.

**Methods:**

Immunohistochemistry was used to detect expression of the inflammation-related protein NOD-like receptor protein 3 (NLRP3) and pyroptosis key protein gasdermin D (GSDMD) in kidney tissues of DKD patients. HK-2 cells were cultured *in vitro* and stimulated with different concentrations of glucose. The changes in HK-2 cell ultrastructure were observed using electronmicroscopy, and western blot was used to detect NLRP3, caspase-1 p20, GSDMD-N, interleukin (IL)-1β, and IL-18 expression.

**Results:**

NLRP3 and GSDMD expression in kidney tissues of DKD patients was higher than that in control subjects. Further, GSDMD expression was positively correlated with that of NLRP3 (*r* = 0.847, *P* = 0.02). After stimulating HK-2 cells for 24 h with different glucose concentrations, compared with the control group, the 15 and 30 mmol/L glucose groups showed typical ultrastructural changes of pyroptosis. The protein expression of NLRP3, caspase-1 p20, GSDMD-N, IL-1β, and IL-18 expression in high glucose group increased significantly compared with the control group, and was glucose-concentration-dependent.

**Conclusion:**

High glucose can activate inflammasome, cause inflammatory cytokines release, and induce pyroptosis in HK-2 cells. NLRP3-caspase-1 may be involved in GSDMD-mediated pyroptosis. This study shows a novel relationship between glucose concentration and pyroptosis, which can be studied further to design better therapies for patients with DKD.

## Introduction

Diabetic kidney disease (DKD) is the primary cause of end-stage renal disease (1). In recent years, it has been found that renal tubular injury and renal interstitial fibrosis are important features of DKD. The micro-inflammatory state is an important basis for the occurrence of DKD (2). In this regard, pyroptosis is an inflammatory programmed cell death mediated by gasdermin D (GSDMD) (3). Inflammasomes are the key to triggering cell pyroptosis. NOD-like receptor protein 3 (NLRP3) is one of inflammasomes, it can sense different pathogen-associated or danger-associated molecular patterns (PAMPs or DAMPs) and oligomerize these components into a very gigantic and efficient protein complexes, leading to Caspase-1 activation and IL-1β and IL-18 production and participate in the regulation of cell functions (4).

In recent years, pyroptosis has become a research hotspot in the field of inflammation globally (5–7). However, there are few studies on the relationship between pyroptosis and DKD. Recently, evidence indicates that inhibition of the NLRP3 inflammasome pathway may reduce the occurrence of pyroptosis in diabetes-related complications (8, 9). However, the specific relationship between NLRP3 inflammasome activation and pyroptosis remains to be clarified. In addition, there have been few experimental studies on whether pyroptosis is involved in the process of renal tubular epithelial cell damage in DKD.

In this study, we compared the expression of pyroptosis-related proteins in human renal tubular epithelial cells and in the kidney tissues of healthy people and patients with DKD. We also used transmission electron microscope (TEM) to observe the occurrence of pyroptosis in human renal tubular epithelial cells treated with different concentrations of glucose, and analyzed the possible mechanism of pyroptosis caused by high glucose concentration. This study may obtain information about the progression of DKD and provide theoretical basis for further study of the molecular mechanism of the pyroptosis-related signal transduction pathway.

## Materials and Methods

### Materials

GSDMD (20770-1-AP) was purchased from Proteintech, China. Dulbecco's modified Eagle's medium/F12 medium, fetal bovine serum, and trypsin were purchased from Gibco, USA. Antibodies to NLRP3 (ab214185), Caspase-1 p20 (ab1872), GSDMD-N (ab215203), IL-1β (ab200478), IL-18 (ab207324), and GAPDH (ab9485) were purchased from Abcam, UK. Goat anti-rabbit (G1213-100UL) secondary antibody and diaminobenzidine chromogenic reagent (G1212-200T) were purchased from Servicebio, China.

### Tissue Specimen Collection

We collected renal tissues from four patients diagnosed with DKD after renal biopsy in the Second Xiangya Hospital of Central South University from December 2019 to May 2020 as the experimental group, and normal renal tissues from three patients undergoing surgical resection after renal trauma as the control group. None of the patients received any immune-related treatment, and all DKD patients were diagnosed by pathology. Informed consent was obtained from all patients prior to tissue sample collection.

### Cell Culture and Experimental Groups

The human renal tubular epithelial cell line HK-2 was from the Nephrology Laboratory of the Second Xiangya Hospital of Central South University; it was cultured with Dulbecco's modified Eagle's medium/F12 medium, 10% fetal bovine serum and 1% double antibiotics (penicillin, streptomycin), and was placed in a cell incubator at 37°C and 5% CO_2_. At the logarithmic growth phase, HK-2 cells were divided into four groups, with the complete medium being replaced with different concentrations of glucose (5.6, 15, 30, and 45 mmol/L), and were cultured for 24 h.

### Biochemical Analyses and ELISA

Routine blood samples for measurement of serum creatinine, eGFR and microalbuminuria were assayed at the central laboratory of Second Xiangya Hospital. Serum NLRP3 was detected by ELISA method.

### Immunohistochemistry

Following gradient ethanol deparaffinization of kidney tissue slices, antigen retrieval was performed, followed by NLRP3 and GSDMD immunohistochemical staining. The slices were incubated overnight at 4°C with primary antibody. Diaminobenzidine staining was performed according to the method specified in the immunohistochemistry kit. In each slice of each group, at least three 200-fold fields of view were randomly selected for imaging; Image J software was used for analysis, and mean density was calculated.

### Transmission Electron Microscope

TEM was used to observe the ultrastructure of cells. The electron microscope fixative solution glutaraldehyde was quickly added after discarding the culture medium for each group. Then, the cells were gently scraped off using a cell scraper and centrifuged. The cells were collected after it was visually observed that they precipitated to the size of sesame seeds or mung beans. New fixative solution was added for fixation, and the cells were dehydrated, embedded in paraffin, and then sliced. The slices were stained with lead citrate to observe the changes in cell ultrastructure.

### Western Blot

The cells were lysed using RIPA lysate buffer containing protease inhibitors. 20 ug protein samples were resolved using 8% SDS-PAGE gel electrophoresis, transferred to polyvinylidene fluoride membrane, blocked using skim milk for 1 h, and incubated overnight at 4°C after adding primary antibodies to detect NLRP3, caspase-1 p20, GSDMD-N, IL-1β, and IL-18. After washing three times with phosphate-buffered saline-Tween 20 (PBST), the membrane was incubated with the secondary antibody [HRP-conjugated goat anti-rabbit IgG (1:5000)] at 37°C for 1 h, then washed and exposured to Kodak film. Using GAPDH protein as an internal reference, the gray-scale ratio between the target protein band and the GAPDH band was semi-quantitatively analyzed using Image Pro Plus software.

### Statistical Methods

The SPSS 23 software was used to perform statistical analysis, and GraphPad Prism 7 software was used for graph plotting. Measurement data are expressed as mean ± standard deviation, and inter-group differences were compared using *t*-test or analysis of variance. Spearman's correlation was used for the analysis, and *P* < 0.05 was considered to indicate statistical significance.

## Results

### Expression of NLRP3 and GSDMD in Kidney Tissues of Healthy People and Patients With DKD

**Figure 1:**
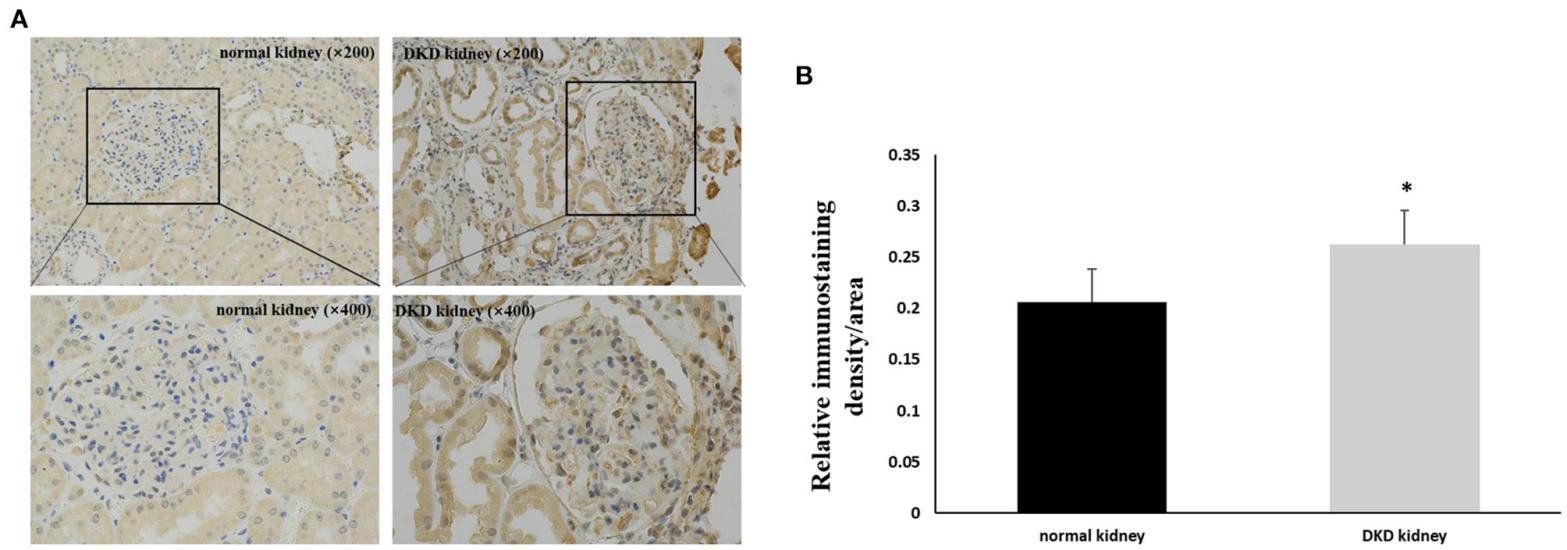
Increased expression of NLRP3 in renal tissues of DKD patients. **(A)** IHC studies revealed increased expression of NLRP3 in DKD patients (×200 and ×400) **(B)** Averaged relative intensity for the staining of NLRP3 in kidney biopsies of normal vs. DKD patients (*P < 0.05).

**Figure 2:**
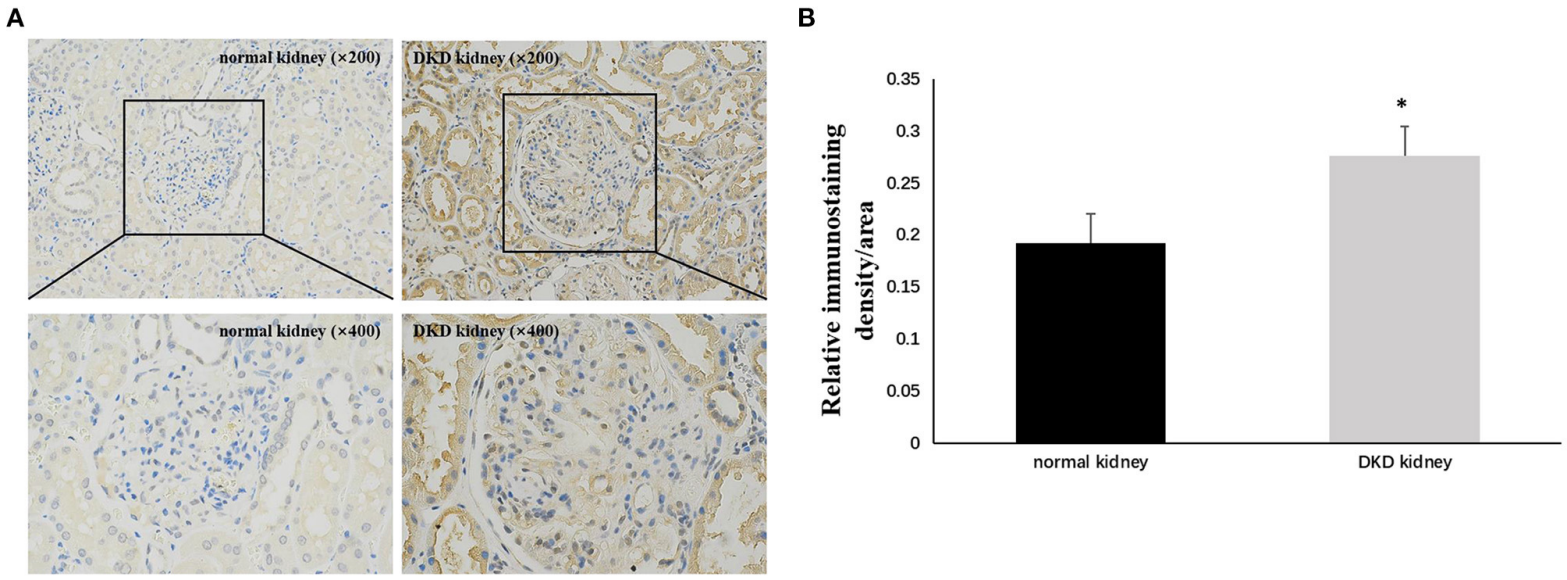
Increased expression of GSDMD in renal tissues of DKD patients. **(A)** IHC studies revealed increased expression of GSDMD in DKD patients (×200 and ×400) **(B)** Averaged relative intensity for the staining of GSDMD in kidney biopsies of normal vs. DKD patients (*P < 0.05).

**Figure 3:**
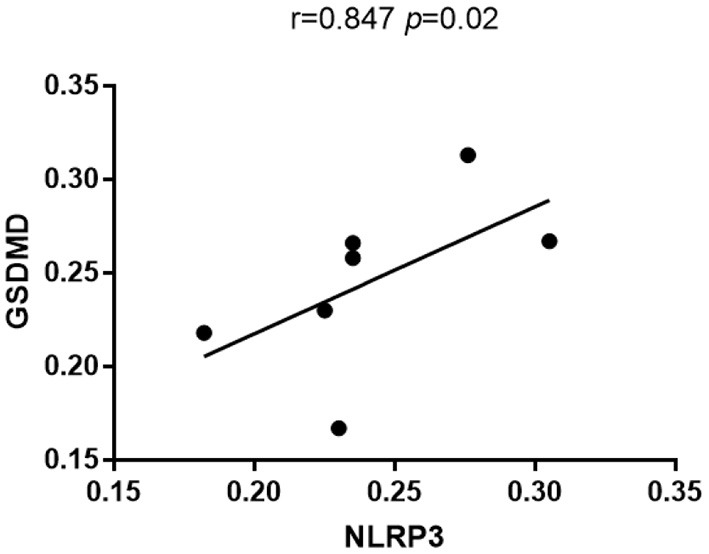
GSDMD expression was positively correlated with that of NLRP3.

Immunohistochemistry results showed that NLRP3 and GSDMD were primarily expressed in renal tubular epithelial cells. NLRP3 was expressed in normal kidney tissue (0.206 ± 0.028); however, its expression in the kidney tissue of patients with DKD was significantly increased (0.263 ± 0.033) (*P* < 0.05; Figure 1). Similarly, GSDMD was also expressed in normal kidney tissue (0.192 ± 0.029), but its expression in the kidney tissue of patients with DKD was significantly increased (0.276 ± 0.028) (*P* < 0.05; Figure 2). Further, GSDMD expression was positively correlated with that of NLRP3 (*r* = 0.847, *P* = 0.02; Figure 3).

### Ultrastructural Changes in HK-2 Cells at Different Glucose Concentrations

**Figure 4:**
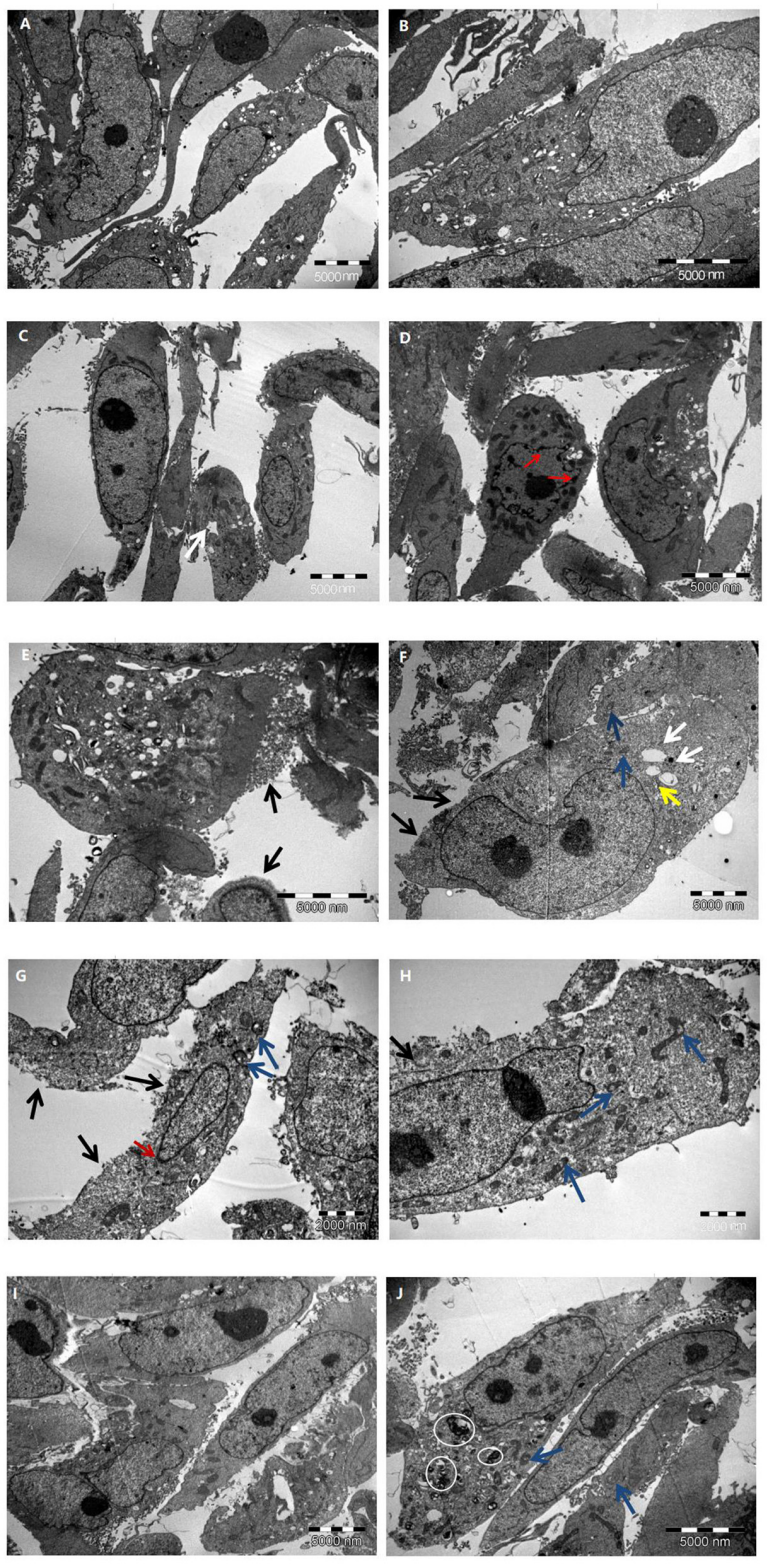
The characteristic of HK-2 cells at different glucose concentrations. **(A,B)** Control group; **(C–E)** 15 mmol/L glucose group; **(F–H)** 30 mmol/L glucose group; **(I,J)** 45 mmol/L glucose group. Black arrow: cell membrane injure; White arrow: endoplasmic reticulum vesiculated expansion; Blue arrow: mitochondrial vacuolation; Yellow arrow: Golgi swelling; Red arrow: chromatin marginated; White circle: myeloid structure.

Different concentrations of glucose (control group 5.6 mmol/L, high glucose groups 15, 30, and 45 mmol/L) were used to stimulate HK-2 cells for 24 h. Using TEM, it was observed that the cells in the control group had an intact structure without swelling; the cell membrane structure was complete and continuous and did not have membranous perforation or cytoplasm content overflow; the cell organelle structure was intact, without swelling, vacuolation, or other damages; the structure of the cell nuclei was complete; and chromatin pyknosis and adhesion to the nuclear membrane was not observed. These observations indicated that the cell structure was normal and no pyroptosis had occurred (Figures 4A,B).

Compared with that in the control group, a higher proportion of cells in the 15 mmol/L group showed slight swelling; some cell membrane edges were fuzzy brush-like, with membranous perforations and cytoplasmic content overflow; some cells showed vesiculation and expansion of endoplasmic reticulum; and the nuclear chromatin showed pyknosis, margination, and adhesion to the nuclear membrane. These observations indicated that a small number of cells in this group had undergone pyroptosis (Figures 4C–E).

Compared with control group, the cell swelling in the 30 mmol/L group was significantly increased; a large number of cell membrane was perforated or had disappeared; a large amount of cytoplasmic content had overflowed; vesiculation and expansion of endoplasmic reticulum, mitochondrial vacuolation, and Golgi swelling were widely present; part of the nucleoli had disappeared; chromatin were marginated and adhered to the nuclear membrane; and the overall electron density of the cells had decreased (cell viability had decreased). These observations indicated that a large number of cells in this group had experienced pyroptosis (Figures 4F–H).

Further, compared with the control group, the 45 mmol/L group showed significant cell swelling; the cell membrane structure was intact and continuous, and did not show membranous perforation or cytoplasmic content overflow; a large number of myeloid structures was observed in the cytoplasm of some cells, showing mitochondrial vacuolation and swelling; there was no significant damage to the cell nuclei, and no phenomena such as chromatin margination were seen. It was revealed that the cells in this group had serious pathological changes, but no significant ultrastructural changes associated with pyroptosis (Figures 4I,J). The comparison of the ultrastructure of HK-2 cells in different group is summarized in Table 1.

**Table 1:** Comparison of HK-2 cell ultrastructure in different experimental groups.

	**Control group**	**15 mmol/L group**	**30 mmol/L group**	**45 mmol/L group**
Cell morphology	Normal	Slight swelling	Significant swelling	Significant swelling
Cell membrane	Intact and continuous	Partial membranous perforation and cytoplasmic content overflow	Membranous perforation and massive cytoplasm content overflow	Intact and continuous
Cytoplasm	The organelle structure was intact without damage	Vesiculation and expansion of endoplasmic reticulum	Vesiculation and expansion of endoplasmic reticulum, mitochondrial vacuolation, and Golgi swelling	Mitochondrial vacuolation and myeloid structure
Nucleus	No chromatin pyknosis or adherence to nuclear membrane	Partial nuclear chromatin pyknosis, margination and adhesion to the nuclear membrane	Disappearance of part of nucleoli, chromatin margination and adhesion to the nuclear membrane	No chromatin margination or adhesion to the nuclear membrane

### Expression of NLRP3 and Pyroptosis-Related Proteins in HK-2 Cells Stimulated by Different Glucose Concentrations

**Figure 5:**
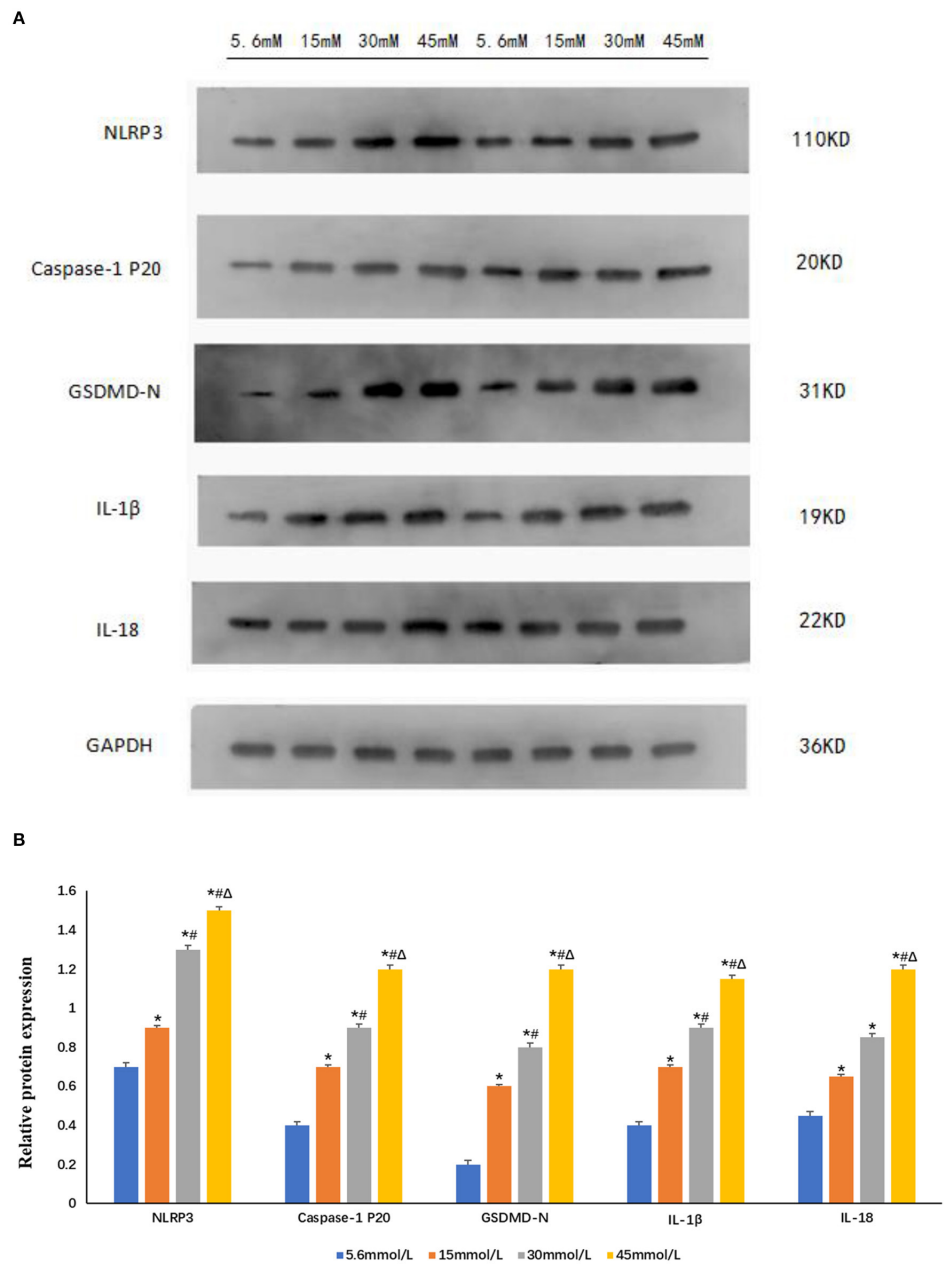
Expression of NLRP3 and pyroptosis-related proteins increased after stimulation with different glucose concentrations in HK-2 cells. **(A)** Western blot showing the expression of NLRP3, caspase-1 p20, GSDMD-N, IL-1β, and IL-18 increased in a glucose-concentration-dependent manner. **(B)** The protein fold change of NLRP3, caspase-1 p20, GSDMD-N, IL-1β, and IL-18, using Gapdh as a reference gene. Data is presented as the average fold change compared to controls ± s.e.m. *n* = 3. *P < 0.05 vs. 5.6 mmol/L glucose group; ^#^*P* < 0.05 vs. 15 mmol/L glucose group; ^Δ^*P* < 0.05 vs. 30 mmol/L glucose group. *n* = 3.

Following stimulation of HK-2 cells with different glucose concentrations (control group 5.6 mmol/L and high glucose groups 15, 30, and 45 mmol/L) for 24 h, we observed that compared with control group, the expression of NLRP3, caspase-1 p20, GSDMD-N, IL-1β, and IL-18 increased significantly in a glucose-concentration-dependent manner (*P* < 0.05; Figure 5).

## Discussion

DKD is the primary cause of chronic kidney disease. Effective treatment of DKD and exploration of new therapeutic targets has always been a major topic of research. In addition to abnormal blood glucose and lipid metabolism, abnormal activation of inflammasomes also plays a key role in the development of DKD (10, 11). Pyroptosis is a form of inflammatory programmed cell death, and NLRP3 inflammasome activation plays a central role in this process. In our study, we found that NLRP3 and GSDMD (key protein of pyroptosis) are primarily expressed in human renal tubular epithelial cells. Their expression in kidney tissue of patients with DKD is significantly higher than that in normal human kidney tissues, and the expression of GSDMD is positively correlated with that of NLRP3. It is therefore speculated that NLRP3 cooperates with GSDMD to participate in the development and progression of DKD.

Inflammasomes are multi-protein complexes assembled by pattern recognition receptors in the cytoplasm. They can recognize a variety of stimuli such as pathogen-associated molecular patterns or damage-associated molecular patterns. By recruiting and activating caspase-1, they induce mature inflammatory factors such as IL-1β and IL-18. NLRP3 is the most widely studied inflammasome, which can be activated through classical or non-classical pathways and participates in the pathogenesis of acute kidney injury and chronic kidney disease (12). The activation of NLRP3 inflammasome in DKD has attracted widespread attention. The activated NLRP3 inflammasome promotes secretion of IL-1β and IL-18, which in turn promotes the progression of DKD (13). Researchers have observed upregulation of NLRP3 and caspase-1 expression in endothelial cells and podocytes in the kidneys of mice and patients with DKD (14). *In vitro* cell culture and animal models have confirmed the role of NLRP3 in DKD (15–17). In this study, we stimulated HK-2 cells with high concentrations of glucose and observed increased expression of NLRP3, caspase-1 p20, IL-1β, and IL-18, suggesting that high glucose concentration activates HK-2 cell inflammasome and causes the release of inflammatory factors. That consistent with previous studies.

Although pyroptosis was initially considered a unique feature of immune cells, recent studies have shown that it also plays a role in non-immune cells (6, 18). In contrast-induced acute kidney injury and renal ischemia-reperfusion injury, renal tubular epithelial cell pyroptosis is an indispensable process (19, 20). The activated NLRP3 activates caspase-1, cleaves GSDMD, breaks self-inhibition, and produces an N-terminal fragment. GSDMD-N targets the cell membrane to form pores and causes water influx, so that the ion gradients across the cell membrane disappear, and the cells undergo swelling and osmotic lysis, eventually leading to cell pyroptosis (21, 22). In our study, we also observed that renal tubular epithelial cells showed obvious pyroptosis with the increase expression of NLRP3 after stimulated by high glucose. In addition, for the first time, we observed through TEM that the HK-2 cells in the high-glucose groups (15 and 30 mmol/L) showed ultrastructural changes that are typical of pyroptosis, such as cell membrane damage, discontinuity, cytoplasmic content overflow, and chromatin margination and adhesion to the nuclear membrane. However, the cells in the control group did not undergo pyroptosis. Further, we found through western blot that compared with that in the normal control group, the expression of NLRP3, caspase-1 p20, GSDMD-N, IL-1β, and IL-18 in the high-glucose groups (15, 30, and 45 mmol/L) increased in a concentration-dependent manner, which also indicated that high glucose concentration promotes the occurrence of cell pyroptosis, and that NLRP3-caspase-1 may be related to GSDMD-mediated pyroptosis. Although the expression of pyroptosis-related proteins in the 45 mmol/L group was higher than that in the other groups, and TEM showed that the cells in this group were significantly swollen with part of cells appearing large number of myeloid structures and vacuolated mitochondria. This phenomenon indicated that the cells were seriously damaged, but there were no ultrastructural characteristics typical of pyroptosis. We speculated that the cells in the 45 mmol/L group have other complex cell damage patterns, such as autophagy.

In conclusion, our study confirmed that high glucose concentration can induce pyroptosis of human renal tubular epithelial cells. We stimulated human renal tubular epithelial cells with different concentrations of glucose, which showed a series of changes in NLRP3 inflammasomes, pyroptosis-related proteins, and inflammatory factors, suggesting that high glucose concentration affects the activation of NLRP3 inflammasome in the autoimmune system, leading to the occurrence of pyroptosis and release of inflammatory factors. This provides a basis for further animal and clinical experiments. With the elucidation of the relevant mechanisms, the targets in the pyroptosis-related signaling pathways are expected to become a new hotspot in the treatment of DKD, and targeting pyroptosis through inflammasome assembly, caspase activation, GSDMD-mediated nuclear pore formation, and other unknown upstream or downstream pathways may be a new way to treat DKD.

## Data Availability Statement

The original contributions presented in the study are included in the article/supplementary material, further inquiries can be directed to the corresponding author/s.

## Ethics Statement

Written informed consent was obtained from the individual(s) for the publication of any potentially identifiable images or data included in this article.

## Author Contributions

FY and YL designed the research. MH and HX did the cell experiment. FY and MH analyzed the data and drafted the manuscript. FY revised the manuscript. All authors have read and approved the manuscript.

## Funding

This work was supported by Grants from the National Natural Science Foundation of China (No. 81770730) and the Natural Science Fund of Changsha (No. kq2014235).

## Conflict of Interest

The authors declare that the research was conducted in the absence of any commercial or financial relationships that could be construed as a potential conflict of interest.

## Publisher's Note

All claims expressed in this article are solely those of the authors and do not necessarily represent those of their affiliated organizations, or those of the publisher, the editors and the reviewers. Any product that may be evaluated in this article, or claim that may be made by its manufacturer, is not guaranteed or endorsed by the publisher.

## Abbreviations

DKD: diabetic kidney disease
NLRP3: NOD-like receptor protein 3
GSDMD: Gasmedermin D
IL: Interleukin
TEM: Transmission electron microscope.
